# Internal Herniation of the Small Bowel Through a Mesosalpinx Defect: GI Image

**DOI:** 10.1007/s11605-021-04935-0

**Published:** 2021-02-09

**Authors:** Carolina Castro Ruiz, Maurizio Zizzo, Valerio Annessi

**Affiliations:** 1 Surgical Oncology Unit, Azienda Unità Sanitaria Locale-IRCCS di Reggio Emilia, Via Giovanni Amendola 2, 42122 Reggio Emilia, Italy; 2 Clinical and Experimental Medicine Ph.D. Program, University of Modena and Reggio Emilia, Modena, Italy

**Keywords:** Mesosalpinx, Broad ligament, Internal hernia, Small bowel obstruction

**Fig. 1:**
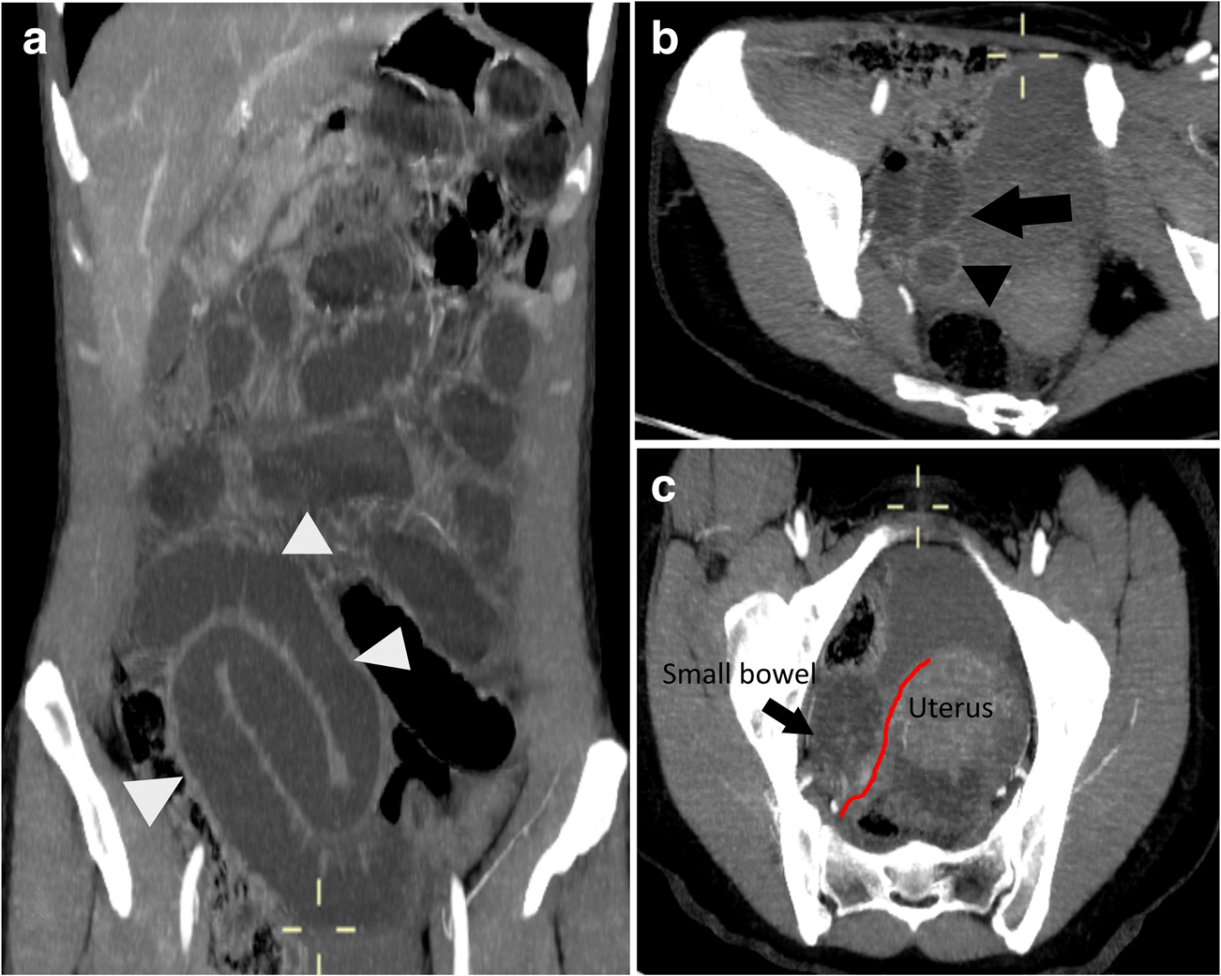
CT scan. (**a**) Coronal CT scan section showing small-bowel dilatation (white arrowheads). (b) Left oblique CT scan section showing a closed-loop image in the right iliac fossa (black arrow) and herniated loop with thickened walls (black arrowhead). (**c**) Transverse CT scan section showing thickening of the broad ligament (red mark)

A 33-year-old Caucasian woman presented to our attention with generalized abdominal tenderness, abdominal distension, and vomiting. Her last bowel movement was 3 days before admission to the emergency room. Past medical and surgical history was unremarkable. On physical examination, abdominal distension with diffuse tenderness on palpation and a reduction of bowel sounds on auscultation were present. Laboratory studies were normal. An abdominal CT scan showed a closed-loop image in the right iliac fossa lateral to the uterine body, dilated small bowel loops containing air-fluid levels, and a fluid collection posterior to the closed loop (Fig. 1).

**Fig. 2:**
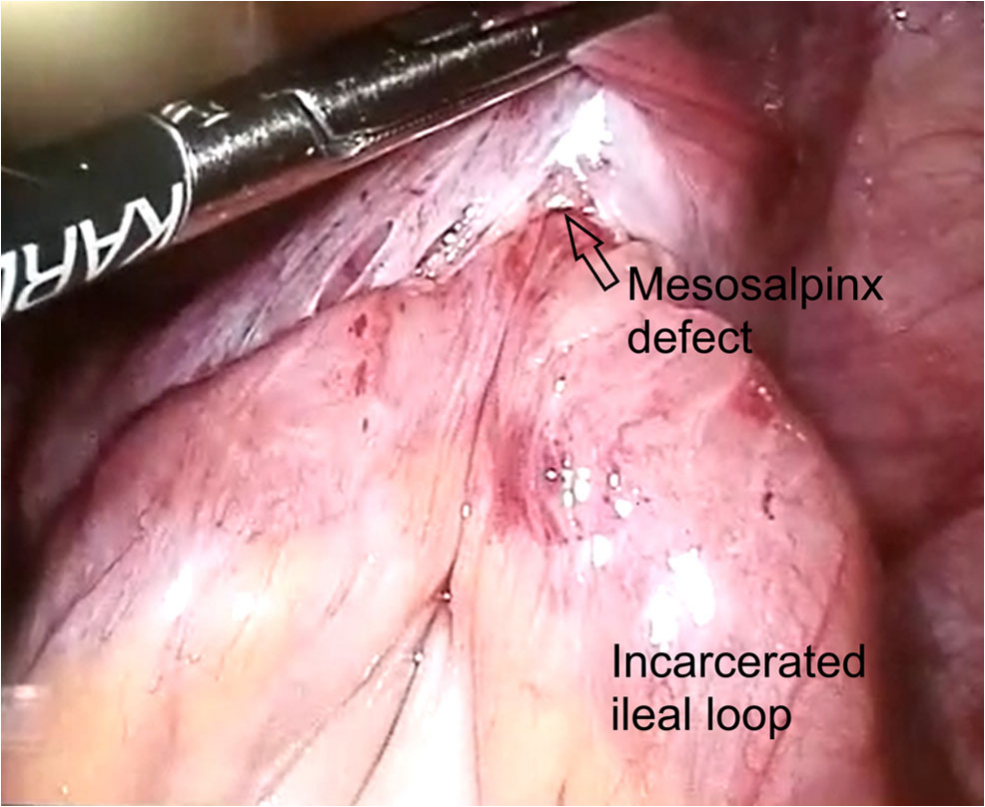
Laparoscopic view of the incarcerated intestinal loop within the right mesosalpinx defect

**Fig. 3:**
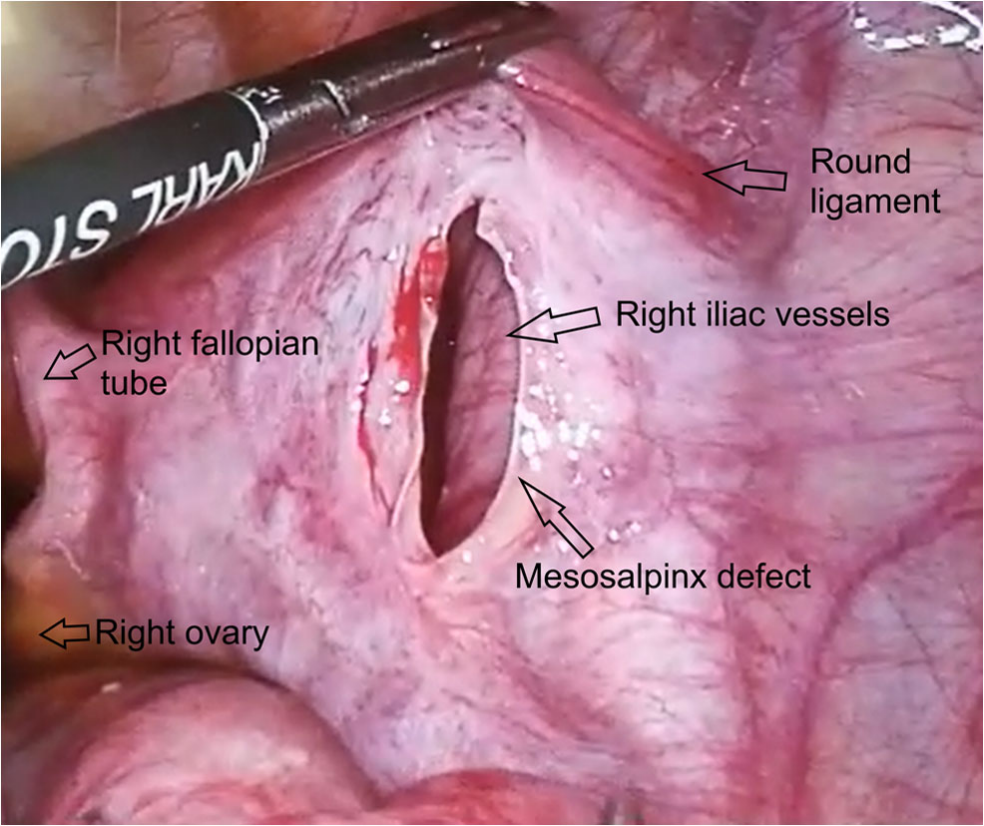
Laparoscopic view of the right mesosalpinx defect after reduction of the incarcerated intestinal loop

Exploratory laparoscopy was performed, identifying the herniation of the penultimate ileal loop through a congenital defect of the right mesosalpinx (Figs. 2 and 3). An enlargement of the mesosalpinx defect by scissors was required to free the irreducible loop. There were no signs of ischemia or tissue damage. The defect was closed with an X 3-0 absorbable stitch. Exploration of the contralateral broad ligament was negative for defects.

The broad ligament of the uterus is a double-layer fold of peritoneum that attaches the lateral portions of the uterus to the lateral pelvic sidewalls; it is divided into mesometrium, mesosalpinx (mesentery of the fallopian tubes), and mesovarium. The presence of a defect on the broad ligament can cause chronic pelvic pain, also known as the Allen-Masters syndrome. This type of hernia is very rare, accounting for 7% of all internal hernias and for 0.9% of all small bowel obstructions (SBO).^1,2^

The pathogenesis of the defects on the mesosalpinx is unknown; it can be congenital or secondary to surgery, pelvic inflammatory disease, or delivery trauma. Clinical presentation may vary from an acute onset with intense abdominal pain due to obstruction and strangulation of the small bowel to a chronic pelvic pain caused by intermittent herniation of the bowel through the defect.^1,2^

Preoperative diagnosis of internal hernia through a mesosalpinx defect can be very difficult, and even though the usefulness of a CT scan has been reported, it does not always make an accurate preoperative diagnosis .^2,3^

Historically the gold standard for treatment of SBO has been open surgery. The first case of internal hernia through the broad ligament treated with laparoscopic surgery was reported by Guillem et al. in 2003 .^3^ Even though laparoscopy has become a standard of treatment in various surgical procedures, its application remains somewhat unclear in the treatment of SBO.

Internal hernias through the broad ligament defects should be taken into consideration as a differential diagnosis in SBO of mechanical nature, particularly in multiparas women with no previous surgical procedures.

Exploratory laparoscopy ought to be kept in mind as a valid tool for treatment and in some cases as a diagnostic tool as well, especially in young women with SBO. During surgery the contralateral broad ligament should always be explored for defects and when present repaired.
